# Implications of KRAS mutations in acquired resistance to treatment in NSCLC

**DOI:** 10.18632/oncotarget.23553

**Published:** 2017-12-21

**Authors:** Marzia Del Re, Eleonora Rofi, Giuliana Restante, Stefania Crucitta, Elena Arrigoni, Stefano Fogli, Massimo Di Maio, Iacopo Petrini, Romano Danesi

**Affiliations:** ^1^ Clinical Pharmacology and Pharmacogenetics Unit, Department of Clinical and Experimental Medicine, University of Pisa, Pisa, Italy; ^2^ Department of Oncology, University of Turin, Ordine Mauriziano Hospital, Turin, Italy; ^3^ General Pathology, Department of Translational Research and New Technologies in Medicine and Surgery, University of Pisa, Pisa, Italy

**Keywords:** KRAS, NSCLC, treatment acquired resistance, TKI, pharmacogenetics

## Abstract

**Rationale:**

KRAS is the most common and, simultaneously, the most ambiguous oncogene implicated in human cancer. Despite KRAS mutations were identified in Non Small Cell Lung Cancers (NSCLCs) more than 20 years ago, selective and specific inhibitors aimed at directly abrogating KRAS activity are not yet available. Nevertheless, many therapeutic approaches have been developed potentially useful to treat NSCLC patients mutated for KRAS and refractory to both standard chemotherapy and targeted therapies.

The focus of this review will be to provide an overview of the network related to the intricate molecular KRAS pathways, stressing on preclinical and clinical studies that investigate the predictive value of KRAS mutations in NSCLC patients.

**Materials and Methods:**

A bibliographic search of the Medline database was conducted for articles published in English, with the keywords KRAS, KRAS mutations in non-small cell lung cancer, KRAS and tumorigenesis, KRAS and TKIs, KRAS and chemotherapy, KRAS and monoclonal antibody, KRAS and immunotherapy, KRAS and drugs, KRAS and drug resistance.

## INTRODUCTION

Lung cancer is the leading cause of cancer-related death worldwide, despite a reduced incidence in western countries and a remarkable improvement in its therapeutic approach. Among lung tumors, Non Small Cell Lung Cancer (NSCLC) is the most common diagnosis and the adenocarcinoma is the predominant subtype [1]. Several driver mutations have been described, in the recent years, in lung adenocarcinomas including those affecting KRAS (15-25%) and EGFR (10-35%). Less commonly AKT1, PIK3CA, HER2, MAPK1, MEK1 and MET mutations have been reported. Moreover, rearrangements involving ALK, ROS1 or RET locus have been identified [2].

The majority of these alterations are targetable by the EGFR tyrosine kinase inhibitors (TKIs) (i.e. gefitinib, erlotinib, afatinib in EGFR-mutant; crizotinib, ceritinib, brigatinib, and alectinib in ALK rearranged tumors) [3–8].

However, there are no specific approved drugs for patients with KRAS mutant tumors and all the anti-KRAS evaluated compounds have failed to demonstrate any clinical activity. Indeed, because of the high frequency of KRAS mutations in NSCLC, several preclinical and clinical investigations have been conducted including inhibition of KRAS protein expression via RNA interference (RNAi), blocking post-translational modification with farnesyl-transferase inhibitors (FTIs) or blocking KRAS localization at the cellular membrane [9–11]. Different strategies evaluated an epigenetic approach, using cyclin-dependent kinases, heat shock proteins or focal adhesion inhibitors [12–14]. Several researchers wagered on inhibitors of downstream effectors of the KRAS signaling pathways (PI3K/AKT/mTOR and RAF/MEK/ERK) without significant success [15–17].

This review focuses on the molecular pathways of KRAS in order to point out possible targets for an anti-KRAS approach reporting success and failures of compounds developed to date.

## MATERIALS AND METHODS

A bibliographic search of the NCBI PubMed database was conducted for articles published in English, using the following keywords: KRAS, KRAS mutations in non-small cell lung cancer, KRAS and tumorigenesis, KRAS and TKIs, KRAS and chemotherapy, KRAS and monoclonal antibody, KRAS and immunotherapy, KRAS and drugs, KRAS and drug resistance.

## RESULTS

### KRAS and its signaling pathways

**Figure 1:**
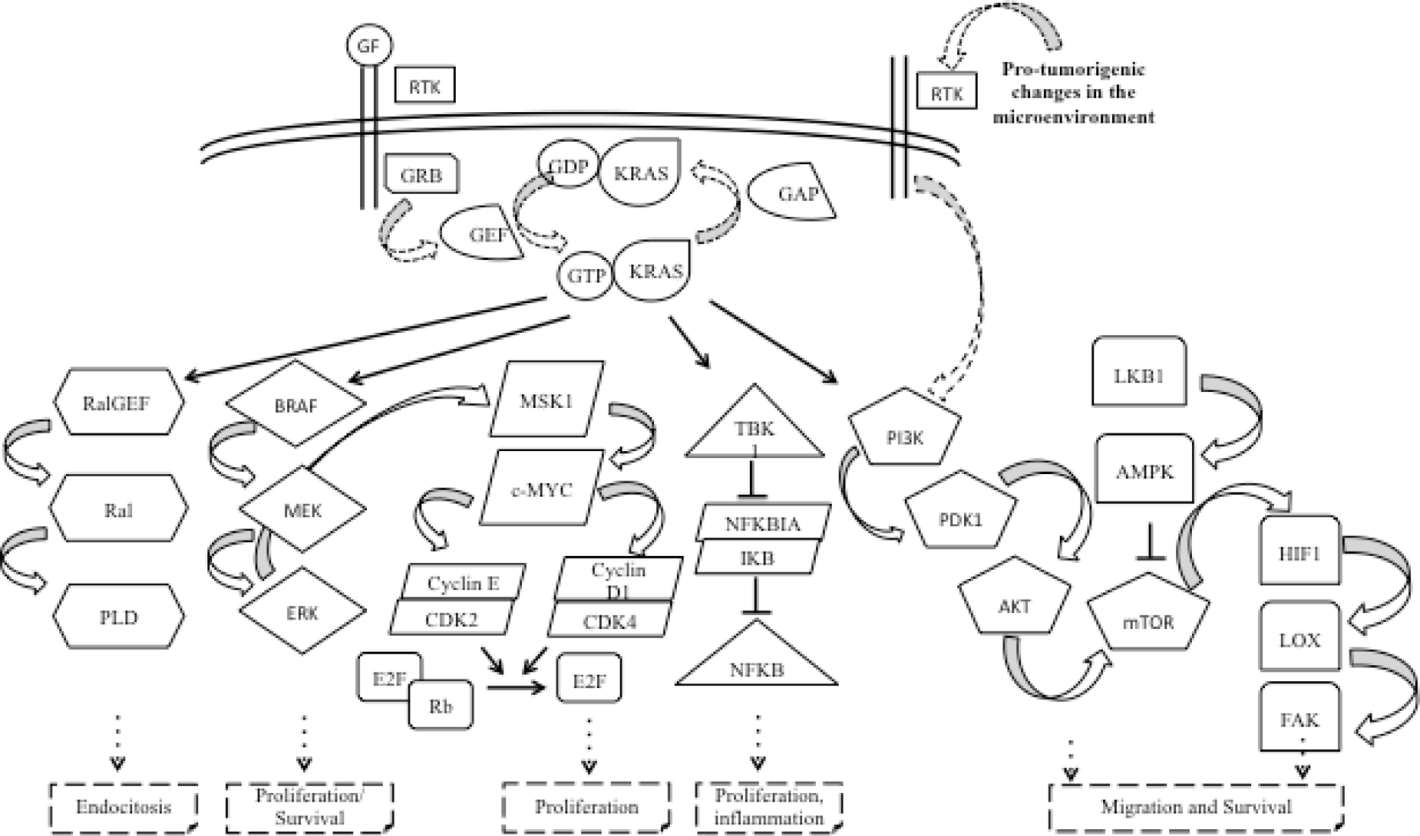
Complexity of KRAS signalling pathways KRAS belongs to the Ras superfamily, a group of small GTP-binding proteins, and serves as a signal transducer from its tyrosine kinase receptors, that are engaged by autocrine and paracrine stimuli. In the active GTP-bound conformation, KRAS activates several effector molecules resulting in endocytosis, cell growth and proliferation, apoptosis, migration, and survival. Abbreviations: GF: growth factor; RTK: receptor tyrosine kinase; GRB: growth factor receptor-binding protein; GEF: guanine nucleotide exchange factor; GTP: guanosine triphosphate; GDP: guanosine diphosphate; KRAS: Ki-ras2 Kirsten rat sarcoma viral oncogene homolog; GAP: GTPase activating protein; RalGEF: RAL-guanine nucleotide exchange factor; PLD: phospholipase D; BRAF: v-Raf murine sarcoma viral oncogene homolog B; Mek: Mitogen-activated protein kinase kinase; Erk: extracellular signal-related kinase; MSK1: mitogen- and stress-activated protein kinase; C-MYC: v-Myc avian myelocytomatosis viral oncogene homolog; CDK2/4: cyclin-dependent kinase 2/4; E2F: E2 transcription factor; Rb: retinoblastoma protein; TBK1: TANK Binding Kinase 1; NFKB: nuclear factor kappa-light-chain-enhancer of activated B cells; NFKBIA: NFKB inhibitor alpha; FAK: focal adhesion kinase; PI3K: phosphoinositide-3-kinase; AKT: v-Akt murine thymoma viral oncogene homolog 1; mTOR: mammalian target of rapamycin; LKB1: serine/threonine kinase 11; AMPK: AMP-activated protein kinase; HIF1: hypoxia-inducible factor 1; LOX: lysyl oxidase; FAK: focal adhesion kinase.

KRAS belongs to a group of small GTP-binding proteins called the RAS superfamily or RAS-like GTP-ases. This group includes Harvey-Ras (H-RAS), neuroblastoma-Ras (N-RAS) and two splice variants of KRAS: KRAS4A and KRAS4B [18]. Following to the binding of growth factors with the respective receptors (i.e. EGFR), KRAS protein bounds to GTP and become able to activate intracellular pathways between a GTP-bound active and inactive state. GTPase activating proteins (GAPs) facilitate GTP hydrolysis, amplifying the intrinsic GTPase activity of KRAS. The interaction with guanine-exchanging/releasing factors (GEFs) promotes the exchange of the GDP with GTP [19]. In the GTP-bound state, KRAS interacts with multiple downstream effectors, including PI3K/AKT/mTOR, RAF/MEK/ERK and Ral-GEF pathways, and regulates cell proliferation, survival, motility, differentiation, endocytosis, angiogenesis, and apoptosis (Figure 1) [20]. Many other signaling pathways are involved in the feedback regulation and crosstalk, which contribute to the complexity of the KRAS signaling network. These pathways include the downstream effector protein kinase Cι (PKCι) involved in tumor-initiating cell phenotype through the PKCι/ELF3/NOTCH3 axis [21]; the EphA2 a receptor tyrosine kinase (RTK) that negatively regulates the KRAS/MEK/ERK pathway after the binding of its ligand ephrin A1 [22]; or the CUB domain–containing protein 1 (CDCP1) and AXL that are two RTKs regulated by many KRAS effectors, like RAF/MEK/ERK and PI3K/AKT/mTOR [23, 24].

### KRAS mutations and NSCLC

KRAS is mutated in 15-25% of NSCLC, mostly adenocarcinoma and occasionally squamous cell carcinoma [25], and is more frequent in white than in Asian populations (25–50% vs 5–15%, respectively) [26]. There is a linkage between smoking habits and KRAS mutations, with an incidence of 25–35% in smokers and only 5% in never-smokers [27–29]. Approximately, 97% of KRAS mutations interest codons 12 and 13 in exon 1, less frequently, mutations occur at codon 61 (Table 1) [30–32]. While these mutations are located near to the GTP binding site, the intrinsic GTP-ase activity is impaired and KRAS accumulates in GTP-bound constitutively active form, which sustains the activation of the KRAS downstream signalling [33].

**Table 1:** Frequency of KRAS mutations in NSCLC

KRAS genotype	Mutation	Frequency (%)	Reference
p.G12C	c.34G>T	42	[25]
p.G12V	c.35G>T	21	
p.G12D	c.35G>A	17	
p.G12A	c.35G>C	7	
p.G13D	c.38G>A	2	

KRAS and EGFR mutations are usually mutually exclusive and, therefore, KRAS mutations can be considered as innate resistance factor for anti-EGFR TKI. However, recent published data, obtained with high sensible technologies, suggest that some tumors can share EGFR and KRAS mutation in a heterogeneous tumor cell population, as well as the co-occurrence of genomic alteration in LKB1 and TP53 [34–39].

### Therapeutic approaches for KRAS-mutant NSCLC patients

#### Inhibition of the KRAS gene expression

One effect of KRAS activation is the induction of telomerase (TERT) transcription [40]. Recently, Liu and colleagues confirmed an increased mRNA expression, telomerase activity and telomere length in lung adenocarcinoma cells with KRAS mutations. However, BIBR1532, a telomerase inhibitor, hampered KRAS-induced cells’ proliferation suggesting that telomerase could represent a promising target in KRAS-mutated NSCLCs [41]. In fact, in a randomized phase II trial, the maintenance treatment with the telomerase inhibitor imetelstat did not improve progression-free survival (PFS) in advanced NSCLC patients [42].

Zhang and colleagues evaluated the anti-tumor effect of an anti-KRAS ribozyme adenoviral vector (KRbz-ADV) in NSCLC cell lines with or without KRAS mutation, finding that KRbz-ADV inhibits significantly the growth of KRAS mutant than wild type cells [43].

Using an RNA interference (RNAi), Sunaga and colleagues in 2011 investigated if the knock down mutant KRAS transcript may revert the malignant phenotype of NSCLC. KRAS expression was inhibited and cell proliferation was reduced alongside with a down regulation of MAPK pathway, however, tumorigenicity was not abolished. These findings remarked the complexity of mutant KRAS oncogenic signaling and cell capability to overcome the KRAS “targeted inhibition” [9]. Despite recent researches encouraged the clinical application of the targeted silencing for NSCLC patients harboring a KRAS mutation [44, 45], further investigations are still needed in the gene therapy field.

### HDAC inhibitors

The histone deacetylase inhibitors (HDACi) block gene transcription, inhibit proliferation and induce apoptosis in tumor cells with promising results for the treatment of some neoplastic proliferative diseases [46]. Kurtze and colleagues evaluated if the treatment of KRAS-mutant NSCLC A549 cell line with vorinostat, an HDACi, could overcome the resistance to the EGFR TKIs [47]. The combination of vorinostat with gefitinib or erlotinib was found to induce apoptosis, revering the TKI-resistance status of A549 cells [47]. Similar results were observed when three NSCLC cell lines, including the KRAS-mutant and EGFR wild type A549, were tested with panobinostat, an HDACi, and erlotinib. As expected, while proliferation of A549 cell line was not inhibited by erlotinib alone, it was impaired by panobinostat treatment, as well as by the synergistic combination of both drugs [48]. In an exploratory biomarker analysis of phase I/II trial investigating the efficacy of gefitinib plus vorinostat in NSCLC, the presence of sensitive EGFR mutations was predictive of higher response rate (RR), longer PFS and overall survival (OS) compared to KRAS mutations [14]. However, these results could be simply related to the high activity of gefitinib in EGFR-mutant patients, and do not clarify the role of HDAC inhibition.

### Inhibition of the KRAS trafficking

**Figure 2:**
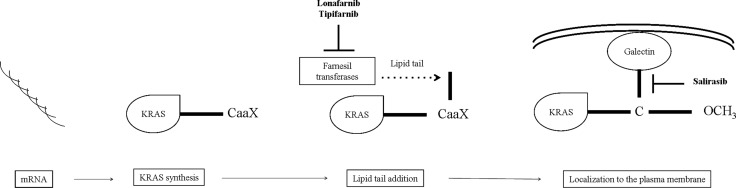
Steps towards KRAS membrane trafficking and localization After KRAS synthesis in the cytoplasm, farnesyl transferases add a lipid tail at a CaaX tetrapeptide motif (C: amminoacid cysteine; aa: two aliphatic residues; X: a variable residue) on the C-terminus of inactive KRAS protein. Lonafarnib and tipifarnib may inhibit this step, interfering with KRAS membrane trafficking. On the other hand, KRAS signaling could be block by salirasib, that targets the localization of KRAS to the membrane. Abbreviations: KRAS: Ki-ras2 Kirsten rat sarcoma viral oncogene homolog; CaaX: carboxyl-terminal.

Post-translational modifications of the KRAS protein is another target for KRAS inhibition. After transduction, KRAS undergoes multi-stage post-translational modifications to become active. First, the protein undergoes prenylation by the addition of a farnesyl tail to its carboxyl-terminal by the farnesyl-transferase (FT-ase) [49]. Several studies have been conducted to determine whether the farnesyltransferase inhibitors (FTIs) have clinical activity in NSCLC patients [10, 50, 51]. Tipifarnib and lonafarnib showed activity *in vitro* and in chemically-induced KRAS-mutant lung tumors in mice [52, 53]. In clinical trials FTIs did not show activity in NSCLC, and they have never been tested in a defined KRAS mutant population [10, 50]. A possible explanation for the FTIs failure may be the presence of an alternative modification, the geranylgeranylation, that is another process to localize protein to the membrane (Figure 2) [54].

### Inhibition of KRAS localization

Other attempts to block the KRAS signalling is to interfere with its localization in cellular membranes using RAS farnesyl cysteine mimetic drugs, like salirasib (farnesylthiosalicylic acid). Mimetic drugs dislodge KRAS from its membrane-anchoring sites and prevent activation of the signaling cascades [54]. Despite promising preclinical data [55], early-phase clinical trials were not successful. Riely and colleagues enrolled 33 patients with stage IIIb/IV lung adenocarcinoma, of which 30 had a KRAS mutation, however, none of the patients raised a radiographic partial response (PR). Despite moderate toxicity (diarrhea, nausea, and fatigue), this phase II trial testing salirasib failed to show any clinical benefit for NSCLC patients harboring KRAS mutations. Interestingly, this was the first trial to examine a targeted therapy specifically in KRAS-mutant NSCLC (Figure 2) [11].

The failure of this trial emphasized the challenges in targeting challenges KRAS prenylation and its membrane localization. First, it is known that an alternative process that could prenylate KRAS proteins exists (geranyl-geranylation). In addition, several signaling molecules are farnesylated (Rho-B, Rho-E, Lamin A, Lamin B, PTP-CAAX1/2), supporting a pleiotropic biological effect, even if KRAS were significantly inhibited by FTIs [49].

### Targeting the downstream effectors of oncogenic KRAS

#### PI3K/AKT/mTOR inhibitors

The PI3K/AKT/mTOR pathway is frequently activated in cancer and maintains tumor growth [56]. In lung cancer, mTOR phosphorylation was found in 51% of NSCLC patients [57]. PI3K/AKT/mTOR pathway is a downstream effector of KRAS and its inhibition could have a role in KRAS mutant NSCLC [58]. Castellano and colleagues reported that PI3K inhibitors cause the regression of KRAS p.G12D-induced benign lung tumors in genetically engineered mouse models [59]. Instead, in mice with malignant lung cancer harboring the KRAS p.G12D, PI3K p.H1047R mutations, and TP53-null, Green et colleagues showed a modest growth inhibition using PI3K inhibitors and little or no survival benefit [60]. Moreover, these results are in line with several clinical observations suggesting a limited activity of PI3K/AKT/mTOR inhibitors in NSCLC. The BASALT-1 trial, evaluating the combination of buparlisib, a PIK3CA inhibitor, with chemotherapy was closed for futility at first interim analysis. The study included 12 patients with KRAS mutation, which had a trend for a better PFS [61].

**Figure 3:**
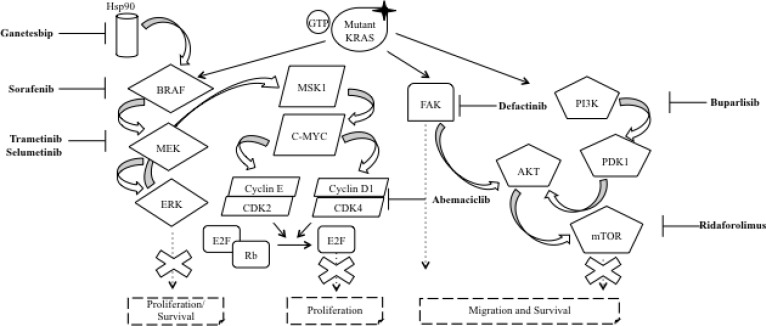
Targeting downstream effectors of oncogenic KRAS In NSCLC, the KRAS protein is often mutated (mutant KRAS) leading to the inactivation of its GTPase activity. The result is the constitutive activation of KRAS and, therefore, of the several effector pathways that are activated downstream of KRAS, with the RAF/MEK/ERK and PI3K/AKT/mTOR as the two pathways that have been studied most in detail. Sorafenib is a multitarget TKI which also inhibits BRAF protein, while trametinib and selumetinib acts against MEK protein. On the other hand, buparlisib and ridaforolimus have been used as PI3K and mTOR inhibitors, respectively. Independently, to these two best characterized pathways, the research focused on the inhibition of other targets. For example, ganetespib, defactinib and abemaciclib act against HSP90, FAK and CDK4, respectively. The goal of these drugs is to stop the tumorigenesis promoted by mutant KRAS. Abbreviations: RTK: receptor tyrosine kinase; Hsp90: heat shock protein 90; GTP: guanosine triphosphate; GDP: guanosine diphosphate; KRAS: Ki-ras2 Kirsten rat sarcoma viral oncogene homolog; BRAF: v-Raf murine sarcoma viral oncogene homolog B; MEK: mitogen-activated protein kinase kinase; ERK: extracellular signal-related kinase; MSK1: mitogen- and stress-activated protein kinase; c-MYC: v-Myc avian myelocytomatosis Viral Oncogene Homolog; CDK2/4: cyclin-dependent kinase 2/4; E2F: E2 transcription factor; Rb: retinoblastoma protein; FAK: focal adhesion kinase; PI3K: phosphoinositide-3-kinase; AKT: v-Akt murine thymoma viral oncogene homolog 1; mTOR: mammalian target of rapamycin.

mTOR inhibitors seem to be able to stop the malignant progression in mice and in preclinical models of NSCLC with a KRAS mutation [62]. However, in the randomized clinical trial, 79 patients with KRAS mutant NSCLC treated with ridaforolimus, only achieved an overall response rate of 1% (Figure 3) [63].

### RAF/MEK/ERK pathway inhibitors

Sorafenib is a multi-tyrosine kinase inhibitor against vascular endothelial growth factor receptor (VEGFR), platelet-derived growth factor receptor (PDGFR) and Proto-Oncogene Tyrosine-Protein Kinase (KIT), competing with ATP in the hydrophobic pocket, leading to the inhibition of MAPK pathway [64]. Smit and colleagues reported in an early phase trial 4 partial responses and 6 stabilisations of diseasein 10 NSCLC patients, harboring the KRAS mutation treated with sorafenib after failure of chemotherapy [65]. In a phase II study, among thirty-seven patients with advanced NSCLC treated with sorafenib, KRAS mutations were found in 11 patients that obtained 60% of disease control rate (DCR) compared to 71% in the KRAS wild type tumors (*p* = 0.69) [66]. The phase II BATTLE trial enrolled patients with advanced NSCLC who had been previously treated with chemotherapy and subsequently experienced a disease relapse [15]. The trial enrolled 255 patients, randomized to one of 4 treatment arms (erlotinib, vandetanib, erlotinib plus bexarotene, or sorafenib) according to their baseline biomarker profile analysis, among which KRAS mutations. The stratified biomarker analysis showed that sorafenib had a higher DCR at 8 weeks in KRAS mutant patients, although the difference was not statistically significant (61% vs. 32%, *p* = 0.11) [15]. Interestingly, Ihle and colleagues pointed out that different types of KRAS mutations may predict for different outcomes: tumors with p.G12C and p.G12V mutations had the worse PFS compared with other codon 12 KRAS mutations or KRAS wild-type (median PFS was 1.84 months vs. 3.55 months vs. 1.95 months, respectively, *p* = 0.046). Moreover, the negative impact of KRAS p. G12C and p. G12V mutations on PFS was particularly evident in patients treated with sorafenib (*p* = 0.026) [67]. The MISSION trial failed in his primary endpoint, and highlighted that there were no differencies in PFS in mutant or not mutant patients for KRAS when treated with sorafenib [16]. Also in the first stage of the BATTLE II trial patients treated with sorafenib (29.5% were KRAS mutant), did not show a difference in DCR compared to wild type [68]. Moreover, several trials evaluated sorafenib in combination with erlotinib for NSCLC patients without any promising results (Figure 3) [69, 70].

### Monotherapy and combinations with MEK inhibitors

Selumetinib is a potent, selective, and ATP-uncompetitive inhibitor of MEK1-2 kinases able to inhibit proliferation of KRAS mutant in NSCLC cell lines [71]. Due to its potency, selumetinib has been tested as either monotherapy or in combination with cytotoxic agents to target KRAS mutant NSCLC patients. In the CUSTOM trial selumetinib was studied in patients with KRAS, NRAS, HRAS, or BRAF mutations. Only one PR was observed in 9 patients treated with selumetinib with a median PFS time of 2.3 months, and a median OS of 6.5 months [72].

Trametinib is a reversible and highly selective allosteric inhibitor of MEK1 and MEK2 kinase activity and has been developed and studied in BRAF-mutant metastatic melanoma [73, 74]. In early phase of clinical development, trametinib has been given to 30 NSCLC patients obtaining only two PR, however, both patients were carrier of a KRAS mutation [75]. Following these results, trametinib was compared to docetaxel in a phase II trial in stage IV KRAS mutant NSCLC [76]. Unfortunately, trametinib did not show its superiority and the study was prematurely terminated.

Overall, these results suggest that the presence of a compensatory signaling pathway makes MEK inhibition not sufficient to achieve a significant antitumor effect. On the other hand, the efficacy of a combined treatment between trametinib or selumetinib and cytotoxic agents in NSCLC cells represented a strong rationale to use this drug combination as a potent strategy to improve treatment response in NSCLC patients with KRAS mutations (Figure 3) [77].

In preclinical models, docetaxel in combination with selumetinib showed an important inhibition of tumor growth and regression [78]. A randomized phase II clinical trial evaluated the combination of docetaxel with selumetinib or placebo, in patients with KRAS-mutant NSCLC that progressed after first-line chemotherapy [79]. Interestingly, patients with KRAS p.G12C or p.G12V mutations treated with selumetinib plus docetaxel showed greater improvement in OS, PFS and ORR compared with the other KRAS mutations [80]. Unfortunately, selumetinib in addiction to docetaxel failed to improve overall survival, progression free survival and overall response rate compared to docetaxel alone in the phase III clinical trial SELECT-1 for KRAS mutant NSCLCs [81].

Trametinib, has been evaluated in a phase I trial in combination with docetaxel or pemetrexed in advanced NSCLC patients with or without KRAS mutations [17]. Both combinations showed activity in KRAS mutant and wild-type tumors. Primary endpoint was the objective response rate (ORR). A confirmed PR was observed in 10 of the 47 patients with NSCLC who received trametinib plus docetaxel (21%). The ORR was 18% (four PRs in 22 patients) in those with KRAS wild-type NSCLC and 24% (six PRs in 25 patients) in those with KRAS-mutant NSCLC. Of the 42 patients with NSCLC treated with trametinib plus pemetrexed, six (14%) had a PR; the ORR was 17% (four of 23) in patients with KRAS-mutated NSCLC versus 11% (two of 19) in KRAS wild-type NSCLC [17]

As far as it is known, KRAS mutant NSCLCs do not respond to EGFR-TKIs treatment [82]. The combination of selumetinib with erlotinib was evaluated in randomized phase II trial and compared with selumetinib alone in KRAS mutant tumors or with erlotinib alone in KRAS wild type tumors [83]. In 41 KRAS mutant tumors, the PFS was 4.0 months (95% CI 2.9–7.8 months) for selumetinib alone and 2.3 months (95% CI 2.0–4.6 months) for the combination; the ORR was 10% and 0%, respectively. This study failed to show improvement in objective response rate or PFS with combination therapy of selumetinib and erlotinib over monotherapy in KRAS mutant and KRAS wild-type advanced NSCLC. Therefore, the authors suggested that further study of selumetinib with erlotinib is not warranted in NSCLC [83].

### CDK inhibitors

Cyclin-dependent kinases (CDKs) are critical proteins for cell cycle’s. Proliferation stimuli, including those through KRAS, converge on the complex CDK4/6 cyclin-D, a critical regulator of the transition between phase G1 and S [84].

CDK4 has been shown necessary for tumor progression in a KRAS-induced lung adenocarcinoma model [85]. Abemaciclib, a CDK4 inhibitor, have shown efficacy in various xenograft models for human cancer including NSCLC [86]. Forty-nine pretreated NSCLC patients were enrolled in a phase I trial to evaluate the safety and the clinical activity of abemaciclib. KRAS mutations were present in 26 patients, while 19 were wild-type (and 4 with unknown KRAS status). Results were encouraging, with a DCR of 54% and 37% in KRAS mutant and wild type tumors, respectively [87]. In another trial, Patnaik et al. enrolled 68 pre-treated NSCLC patients for treatment with abemaciclib, including 29 patients harboring KRAS mutation. Even if responses were uncommon, the majority of tumor regression occurred in KRAS-mutant patients, with a stable disease as result [12]. The phase III trial JUNIPER is currently evaluating abemaciclib versus erlotinib in pretreated patients with NSCLC harboring KRAS mutations (Figure 3) [88].

### Hsp90 inhibitors

Heat Shock Proteins (HSPs) are adenosine triphosphate (ATP)–dependent chaperones with an important role for the cell response to stress and for maintaining cellular homeostasis. The ubiquitously expressed HSP90 has been studied intensively because of its involvement in the folding, stability and function of several oncogenic driver proteins. Interestingly, HSP90 chaperone is necessary for the maturation of proteins involved in KRAS downstream pathways, such as mTOR and MEK [89]. Hence, this chaperone could be an attractive therapeutic target for KRAS mutant NSCLC, and several HSP90 inhibitors have been developed for the treatment of cancer. KRAS mutant NSCLC cell lines were sensitive to some HSP90 inhibitors, such as tanespimycin, alvespimycin or ganetespib [90, 91]. Ganetespib has been tested in a phase II study, in stage IIIB/IV NSCLC patients [92]. Unfortunately, this trial failed to demonstrate a significant activity of ganetespib with respect to KRAS mutant NSCLC [92]. Preclinical evidences suggest a synergistic effect of taxanes and ganetespib [93] and in the randomized phase II trial GALAXY-1, docetaxel was administered with or without ganetespib in pretreated NSCLC patients [13]. However, in the KRAS mutant population, the combination did not improve the PFS neither the OS [13], and these data were confirmed in the phase III trial GALAXY-2 (Figure 3) [94].

### Immunotherapy for KRAS-mutated NSCLC patients

Recently, the attention has turned into the discovery of drugs able to interfere with specific immune checkpoints, among which programmed death-1 receptor (PD-1) and its ligand (PD-L1) have been the most studied in NSCLC. PD-1 and PD-L1 are expressed by activated immune cell types, including T-cells, B-cells, dendritic cells, and their interaction negatively regulates immune activity in peripheral tissues in response to infection or tumor progression [95]. Several studies have shown that PD-1/PD-L1 pathway is manipulated by cancer microenvironment, in particular PD-L1 is commonly up-regulated in NSCLC and PD-1 is expressed on the majority of tumor-infiltrating immune cells [96, 97].

FDA has recently approved nivolumab an anti-PD-1 antibody, for metastatic NSCLC progressed after prior platinum-based chemotherapy [98]. Subsequently, pembrolizumab, another anti-PD-1 antibody, was approved as a second-line for NSCLC patients whose tumors exhibit >50% of PD-L1 expression [99] and as first-line treatment in patients PD-L1 positive, with no EGFR or ALK genomic tumor aberrations [100, 101].

However, PD-L1 is clearly not an easy-to-handle biomarker. In fact, different assays are used to determine its expression levels, with different cut-off of PD-L1 positivity making difficult the definition of a strong cut-off value for PD-L1 positivity in NSCLC.

Interestingly, several data report that a high mutational burden is a positive predictive biomarker of response to immunotherapy [102]. For this reason, an increasing number of studies are exploring the incidence of PD-L1 expression with other genetic alterations [103, 104]. The relationship between the KRAS status and PD-1/PD-L1 expression is currently not enough defined in the subset of NSCLC tumors, with also different results [105–108]. However, the subgroup analysis of the CheckMate 057 trial reported an increased effect in terms of OS in favor of nivolumab for KRAS mutant patients, compared to those with not detected or not reported status [98]. In a retrospective study, the expression of PD-1 and PD-L1 has proved to be heterogeneous within KRAS mutant NSCLC, suggesting PD-L1 expression is not genetically driven by KRAS mutation [109]. D’Incecco et colleagues hypothesized that PD-1/PD-L1 expression could differ according to the molecular phenotype of the tumor [106]. Results of their study confirmed that patients harboring KRAS mutations had higher levels of PD-1 expression when compared to the KRAS wild-type population. By contrast, PD-L1-positive tumors showed driver mutations, as EGFR mutations and ALK rearrangements [106]. Conversely, a recent study found a statistical significance between PD-1 expression and KRAS status (*p* = 0.043) and a higher mutation rate in patients with lower PD-1 expression (8 out of 10 KRAS mutant patients); whereas PD-L1 expression was higher in patients harboring EGFR mutations or with wild-type KRAS status [110]. Moreover, according to the study by Zhang et al, mutational status of KRAS correlated with PD-L1 expression was not significantly associated with relapse-free survival and OS [107]. Scheel and colleagues demonstrated a strong association between mutations in KRAS with PD-L1 expression in adenocarcinoma specimens (OR = 2.5; *p* = 0.018) [111].

Recently, according to findings from the phase III OAK trial, atezolizumab, an anti-PD-L1 antibody, improved survival compared with docetaxel in NSCLC patients following the failure of platinum-based chemotherapy, regardless of PD-L1 expression or histology. Even if the number of patients with KRAS mutations was small, the benefit was consistent also across this subgroup [112].

Concerning the association between KRAS mutations and the response to anti-PD-1 antibodies, results of published studies are still limited and controversial.

To conclude, the results for immune checkpoint inhibitor in NSCLC are encouraging, however, a multitude of questions related to predictive biomarkers to anti-PD-1/anti-PD-L1 therapies remains unanswered.

A new hypothesis suggests that the extreme mutational heterogeneity of a tumor, such as in KRAS mutant NSCLC [113], may play a critical key role in the tumor susceptibility to checkpoints blockers by springing an intense immune response against neoantigens and, consequently, improving the response to immune checkpoint-targeting therapies [114]. Even if this concept would be difficult to introduce into daily clinical practice, it is a charming possibility that should be evaluated in future clinical trials.

### KRAS and TKIs resistance

In a recent study on cell-free circulating tumor DNA (cftDNA), Del Re and colleagues evaluated the appearance of KRAS mutations in EGFR positive NSCLC patients progressed after a TKI regimen. Patient that developed a KRAS mutation showed a worse survival compared to KRAS wild-type patients, suggesting a role of KRAS mutations also in acquired resistance to anti-EGFR TKI [37]. Guibert and colleagues obtained similar results, highlighting the correlation between the presence of KRAS mutations at codon 12 with a poor response to therapy [115]. Chabon and colleagues observed resistance mechanisms in 46% of 43 NSCLC patients treated with the third-generation epidermal growth factor receptor (EGFR) inhibitor rociletinib. Again, three patients showed acquiring activating mutations in KRAS following treatment with rociletinib [116]. In two recent published case reports was reported the concomitant presence of EGFR, KRAS mutations and the c-ROS oncogene 1 (ROS1) rearrangement in one NSCLC patient and the concomitant presence of ALK rearrangement and KRAS mutations in other two. Interestingly, these patients experienced a rapid disease progression and primary resistance to crizotinib [36, 117]. Moreover, in 9 out of 16 NSCLC patients, KRAS mutations p.G12D or p.G12V appeared in cftDNA at the time of resistance to ALK-TKIs (crizotinib or ceritinib) and 3 of them presented simultaneously ALK mutations [118].

## CONCLUSIONS

Several preclinical and clinical investigations have been launched with the hope to better understand the biologic world that surround the KRAS gene, its potential prognostic and predictive role and, importantly, to look for effective treatments for NSCLC patients harboring KRAS mutations. However, KRAS targeting seems to be a real challenge to overcome, a direct KRAS-targeting is probably not efficacious because it is able to activate multiple mechanisms of escape under the selective pressure of treatments. One of the best pharmacological rational approach could be based on a combination of treatments, in order to silence more that one driver at the same time. However, the toxicity-side in this case plays an important role and needs to be strongly considered.

Tumor heterogeneity increases the complexity of the system [119]. Acquired mutations, including KRAS, can affect tumor growth due to the evolution of sub-clones that evolve through the selection of advantageous driver, neutral “passenger” or deleterious mutations [120]. This dynamic diversity is the most important mechanism of acquired resistance to treatments.

Therefore, the landscape is becoming complex considering that KRAS mutations can concomitantly occur with two or more driver alterations in the same tumor.

Although we are fully aware of the laborious path that leads the scientific community to face this challenge, we are confident that some of the attempts discussed in this contest could be an effective treatment for KRAS mutant NSCLC patients, and we strongly believe that in this landscape a multidisciplinary approach can help in the management of this complex disease [121].
